# Mood Modulates Auditory Laterality of Hemodynamic Mismatch Responses during Dichotic Listening

**DOI:** 10.1371/journal.pone.0031936

**Published:** 2012-02-22

**Authors:** Lisa Schock, Miriam Dyck, Liliana R. Demenescu, J. Christopher Edgar, Ingo Hertrich, Walter Sturm, Klaus Mathiak

**Affiliations:** 1 Department of Psychiatry, Psychotherapy and Psychosomatics, Medical School, RWTH Aachen University, Aachen, Germany; 2 Interdisciplinary Centre for Clinical Research, Medical School, RWTH Aachen University, Aachen, Germany; 3 Jülich-Aachen Research Alliance (JARA)-Translational Brain Medicine, Jülich, Aachen, Germany; 4 Department of Radiology, The Children's Hospital of Philadelphia, Pennsylvania, United States of America; 5 Hertie Institute for Clinical Brain Research, Department of General Neurology, University of Tübingen, Tübingen, Germany; 6 Department of Neurology, Clinical Neuropsychology, Medical School, RWTH Aachen University, Aachen, Germany; 7 Institute for Neuroscience and Medicine, INM-1, Research Centre Jülich, 52425 Jülich, Germany; Baycrest Hospital, Canada

## Abstract

Hemodynamic mismatch responses can be elicited by deviant stimuli in a sequence of standard stimuli even during cognitive demanding tasks. Emotional context is known to modulate lateralized processing. Right-hemispheric negative emotion processing may bias attention to the right and enhance processing of right-ear stimuli. The present study examined the influence of induced mood on lateralized pre-attentive auditory processing of dichotic stimuli using functional magnetic resonance imaging (fMRI). Faces expressing emotions (sad/happy/neutral) were presented in a blocked design while a dichotic oddball sequence with consonant-vowel (CV) syllables in an event-related design was simultaneously administered. Twenty healthy participants were instructed to feel the emotion perceived on the images and to ignore the syllables. Deviant sounds reliably activated bilateral auditory cortices and confirmed attention effects by modulation of visual activity. Sad mood induction activated visual, limbic and right prefrontal areas. A lateralization effect of emotion-attention interaction was reflected in a stronger response to right-ear deviants in the right auditory cortex during sad mood. This imbalance of resources may be a neurophysiological correlate of laterality in sad mood and depression. Conceivably, the compensatory right-hemispheric enhancement of resources elicits increased ipsilateral processing.

## Introduction

Laterality effects can emerge as a function of emotional state [1]–[4]. According to the right-hemisphere hypothesis, the right hemisphere is dominant in the processing of emotions [5], [6]. According to the valence hypothesis, the right hemisphere is specialized for processing negative valence and the left hemisphere for processing positive valence [7], [8]. Recent work on this topic showed that these two approaches complement each other and reflect different aspects of emotion processing [9], [10]. In particular, the approach-withdrawal model states that right frontal regions mediate withdrawal behavior (for a review see [11]). A lack of positive affect and approach behavior can be observed in depressive disorder and is associated with a relative decrease of left frontal activation.

Laterality effects have been observed in affective disorders and related to the processing of emotion [4], [12], with left hemifield stimuli yielding reduced processing as compared to right hemifield stimuli in depression. Liotti and Mayberg [4] suggested that limbic activation in transient sadness and depression leads to a down-regulation of cortical areas such as inferior parietal and dorsolateral prefrontal cortex in the right hemisphere. Moreover, frontal cortical sites are involved in the regulation of mismatch responses in the auditory cortex [13]–[15] and belong to a combined network for alertness and spatial attention [16], [17]. Schönwiesner and colleagues [15] suggested that temporal regions are involved in the detection and detailed analysis of change, whereas the prefrontal cortex activation may be due to the allocation of attention resources to novel stimuli. A modulation of these prefrontal areas in the right hemisphere in induced sad mood may therefore influence auditory cortex activation to deviant sounds. Similarly, in previous studies, increased processing of right-ear stimuli in depressive disorder was observed [18], [19]. These studies, however, used dichotic listening paradigms that required explicit answers of the subjects. Pre-attentive measures enable the performance of mood induction tasks without interference.

Several studies have investigated the effects of emotional context on pre-attentive processing of auditory stimuli with electrophysiological measures [20]–[22]. Alexandrov and colleagues [20] created an emotional context by monetary reward or punishment and reported significantly larger auditory cortex event-related potentials in response to negative as compared to positive trials. In an fMRI study by Domínguez-Borràs and colleagues [23], subjects conducted a color decision task embedded in the presentation of facial expressions of negative and neutral valence. In the context of negative expressions, responses to novel sounds in superior temporal gyrus were enhanced as well. The effects of emotional context on pre-attentive processing of *lateralized* auditory stimuli in the healthy brain, however, are largely unknown. We hypothesized that ongoing emotion processing in frontal cortices can elicit an imbalance of processing resources with right auditory cortex showing reduced activation, resulting in enhanced processing of deviant sounds at the right ear.

The present study investigated the influence of induced mood on laterality in the processing of neutral language stimuli. Using a design that elicited the hemodynamic analogue of the mismatch negativity (MMN), the influence of different mood states on the processing of unattended dichotically presented acoustic stimuli at auditory cortices was examined. Specifically, mood induction in healthy volunteers was achieved by showing emotional facial expressions (sad, happy and neutral expressions, respectively) [24], while subjects were simultaneously presented a dichotic oddball sequence with consonant-vowel syllables. The task-irrelevant oddball design provided the possibility of investigating laterality effects without disturbing the mood induction procedure. Hemodynamic responses to deviant stimuli were expected within the superior temporal plane. Furthermore, distinct activation patterns should be associated with the different emotion conditions, e.g., sad mood exhibiting right-lateralized prefrontal involvement. As concerns the interaction of mood with the dichotic processing we hypothesized that sad mood would give rise to a relative increase of activation to right-ear deviants due to the interference with the auditory processing in the right hemisphere.

## Materials and Methods

### 2.1 Subjects

Twenty healthy volunteers (age 20–32 years) participated in the study. All subjects were right-handed, as indicated by the laterality quotient (mean 88.2±13.5) of the Edinburgh Inventory [25]. An intelligence screening was included to better describe characteristics of the sample [26]. All participants were native German speakers and had no history of neurological or psychiatric illness. Subjects were screened with the Structured Clinical Interview (SCID-I, [27]) for the Diagnostic and Statistical Manual of Mental Disorders (DSM-IV) to exclude subjects with a psychiatric disorder. Acute medical conditions under pharmacological treatment were excluded; one male participant reported intake of cholesterol-lowering drugs (statins), three of the seven female participants were taking oral contraceptives. Subjects were students or employees of the RWTH Aachen University (see Table 1 for demographic characteristics of the sample). The study was approved by the local Ethics Committee of the Medical School of the RWTH Aachen University and was performed in accordance with the Code of Ethics of the World Medical Association (Declaration of Helsinki). Written informed consent was obtained prior to participation in the study.

**Table 1:** Demographic characteristics of the sample (mean±SD).

	Age	Gender (female/male)	Education (A-levels/university degree)	Verbal intelligence (MWT-B) [N = 16]
N = 20	25.5±2.9	7/13	13/7	119.4±12.3

MWT-B: The Multiple-Choice Vocabulary Intelligence Test [Der Mehrfachwahl-Wortschatz-Intelligenztest, 26]; verbal intelligence screening asking participants to find existing German words among non-words in a multiple-choice task.

### 2.2 Stimuli

#### 2.2.1 Dichotic stimuli

Auditory stimuli were consonant-vowel syllables /ba/, /da/, /ga/, /ka/, /pa/, and /ta/, each recorded twice to allow for a stereo effect when composing the dichotic stimuli, i.e., even the stimuli with the same syllable presented to each ear were not perceived as ‘inside the head’ [28]. Stimuli were adjusted with respect to amplitude and duration. All 36 dichotic combinations of the six CV syllables were presented in a behavioral task, whereas the fMRI experiment only used dichotic combinations of the three syllables /ba/, /pa/, and /ga/.

#### 2.2.2 Emotional face stimuli

For mood induction, 72 color photographs of actors expressing sadness, happiness or neutral emotion from a standardized stimulus set were selected [29]. This standardized face-battery has been proven an effective tool for inducing different mood states [24]. No actor appeared more than once within a session, and faces were balanced for gender.

### 2.3 Dichotic listening behavioral task

A dichotic listening pretest for determining laterality was conducted outside the fMRI scanner. Every dichotic combination of the six different syllables /ba/, /da/, /ga/, /ka/, /pa/, and /ta/ (6×6 = 36) was presented ten times. Of these 360 stimuli, 300 items represented dichotic pairs composed of two different syllables. Subjects were asked to identify the most salient percept of each dichotic pair and indicated their answer in written form by choosing one of the six syllables in a 6-alternatives forced-choice task.

### 2.4 Mood induction procedure

**Figure 1:**
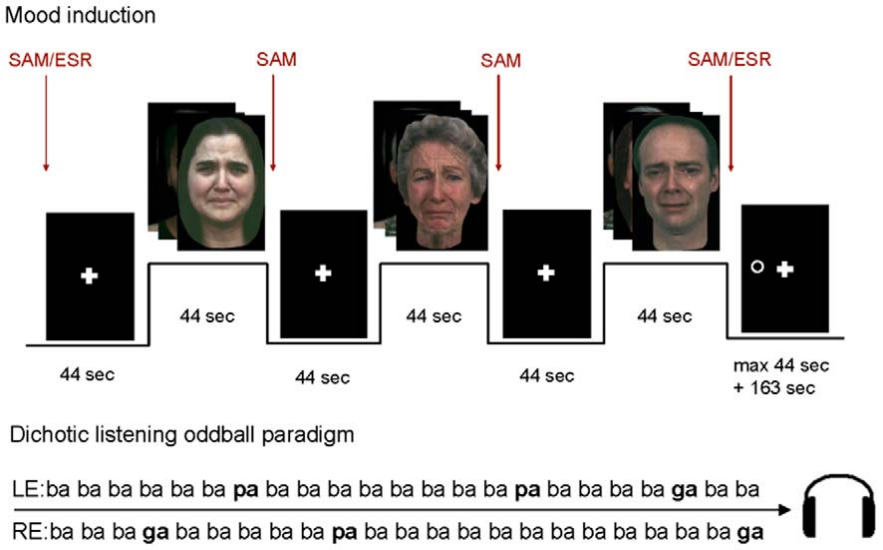
Scheme of an experimental run inducing sadness. A dichotic oddball paradigm was presented to elicit pre-attentive auditory processing during the mood induction task. A visuospatial attention task was added to ‘wash out’ induced mood and attention lateralization prior to the next run; SAM: Self-Assessment Manikin, ESR: Emotional Self-Rating, LE: left ear, RE: right ear.

Mood induction was carried out in a blocked fMRI design (Figure 1, [24]). Sad mood, happy mood, and neutral mood were induced by instructing the subjects to look at the faces and feel the emotion they perceived. The entire session was subdivided into six mood induction runs. Each run was assigned to a single target mood, resulting in two runs per emotion across the entire experiment. The order of the mood induction runs was randomized and balanced across subjects. Within each run, emotions were presented in three blocks of mood induction, with each block preceded by a resting baseline (display of a fixation cross). Emotional block duration was 44 seconds, comprising eight facial stimuli shown for five seconds each in addition to the SAM ratings (Self-Assessment Manikin, SAM; [30]). The order of stimuli was counterbalanced within every run.

### 2.5 Mood ratings

Two types of mood ratings were applied [24]. An explicit verbal rating required subjects to indicate the intensity they experienced the emotions happiness, sadness, anger, fear, disgust, and neutrality on a 6-point scale (Emotional Self-Rating, ESR; [31]). The second rating was a non-verbal rating on a visual 5-point scale. Subjects rated the perceived valence and arousal from 1 = very negative/weak to 5 = very positive/strong (SAM). Prior to the first mood induction block, subjects were asked to perform both ratings. After the first and second block, subjects indicated their arousal and valence on the SAM rating only. After the third mood induction block, both ESR and SAM were completed again (see Figure 1).

### 2.6 Visuospatial attention task

After the last rating, a visuospatial attention task was presented. A small circle was randomly presented to the left or right visual field with a stimulus duration of 100 ms, a fixation duration of 800 ms, and a variable inter-trial interval of 2,500–4,000 ms. Subjects were instructed to fixate on the cross in the middle of the screen and to press a button as fast as possible when the circle appeared. Due to technical limitations, button responses were not recorded. The task was administered in order to involve bilateral attention resources and, as such, to ‘wash-out’ mood state and attention shifts prior to the next run. For fMRI, this time period was modeled as a nuisance variable.

### 2.7 Dichotic stimulation procedure

A task-irrelevant oddball sequence with dichotic stimuli was administered simultaneously to the mood induction run, with a stimulus onset asynchrony of 667 ms (i.e., 3 stimuli per repetition time [TR = 2 sec.]). The auditory oddball sequence consisted of frequent CV syllable /ba/ (different types of /ba/-recordings at the left and right channels: /baL/-/baR/). In the 10% deviants (2.5% /pa/ and 2.5% /ga/ at the left and the right channel, respectively), the contralateral /ba/ items were identical within the respective channel of the frequent stimuli (e.g., /baL/-/ga/ or /ga/-/baR/). Subjects were instructed to ignore the sounds and only pay attention to the mood induction procedure. This experimental design allowed examining the allocation of bottom-up controlled spatial attention without interfering with the mood induction task. Three different oddball sequences were prepared in advance and one assigned to each of the three mood conditions. This assignment was kept for the entire experiment. With the fourth image acquisition after three dummy scans, the oddball sequence started and lasted until the last image acquisition of the run.

### 2.8 fMRI data acquisition

Scanning was performed on a 3 T Magnetom Trio MR scanner (Siemens Medical Systems, Erlangen, Germany) in the department of Psychiatry, Psychotherapy and Psychosomatics at the Medical School of the RWTH Aachen University. Functional images were collected with echo planar imaging (EPI) sensitive to blood oxygenation level dependent (BOLD) contrast (interleaved acquisition of 34 slices, TR = 2,000 ms, echo time [TE] = 28 ms, flip angle [FA] = 77°, slice thickness = 3 mm, gap 0.75 mm, matrix size = 64×64, field of view [FOV] = 192×192 mm^2^, voxel size = 3×3 mm^2^). Slices covered the entire cerebral cortex and were positioned oblique-transversally to achieve maximal brain coverage. Two hundred and thirty volumes were collected per session. The first three volumes of each session were excluded to remove the influence of T1 saturation effects. Head movement was minimized with the use of foam wedges to securely hold the head in the 12-channel head coil. Structural images were obtained using a high-resolution T1-weighted 3-D sequence (TR = 1,900 ms; inversion time [TI] = 900 ms; TE = 2.52 ms; FA = 9°; FOV = 256×256 mm^2^; 176 3D-partitions with an isotropic resolution of 1 mm).

### 2.9 fMRI procedures

Visual stimuli were presented via MR-compatible video goggles and dichotic stimuli were presented through MR-compatible headphones with about 30 dB attenuation of the environmental noise (VisuaStimDigital, Resonance Technology, RT, Northridge, CA, USA). Earplugs further reduced scanner noise. The volume of the auditory stimuli was individually adjusted to a comfortable listening level and good audibility during scanner noise.

### 2.10 Analysis of behavioral data

#### 2.10.1 Dichotic listening task

A laterality index was computed by subtracting left-ear decisions from right-ear decisions in the 300 pairs of lexically different syllables (all other choices were excluded). Laterality indices larger than zero indicated a right-ear advantage (REA). A group mean of right-ear minus left-ear decisions was computed.

#### 2.10.2 Mood ratings

SAM ratings were analyzed for arousal and valence separately, with repeated-measures ANOVAs conducted with the factors mood and timepoint of rating (3×4). Significance level was set at p<.05 and then Bonferroni-corrected in pairwise comparisons. Ratings of the ESR were analyzed on a descriptive level [24], displaying the emotion rated highest in the three mood induction conditions prior to and after the mood induction blocks.

### 2.11 Analysis of fMRI data

fMRI data analyses were calculated using Statistical Parametric Mapping software (SPM8; www.fil.ion.ucl.ac.uk) implemented in MATLAB (TheMathWorks, Natick, MA, USA). After discarding the first three volumes, 227 volumes from each participant were spatially realigned to the mean image to correct for head movement. The next step was normalization into the stereotaxic anatomical MNI (Montreal Neurological Institute) space with 2 mm isotropic voxels. The normalized data were spatially smoothed with an 8 mm isotropic Gaussian kernel to account for inter-subject variability in brain anatomy and to increase signal-to-noise ratio.

The experimental conditions were modeled in a mixed blocked and event-related design convolved with the canonical hemodynamic response function (hrf) and its temporal derivative for a more differentiated modeling of the time course. The design comprised six sessions (2×3 emotions: sad, happy, neutral) with three mood induction blocks per session and the deviant syllables as events. The following regressors were modeled for each session: instructions, ratings, mood induction blocks, visuospatial task, deviant syllable /ga/ presented to the left ear, deviant syllable /pa/ presented to the left ear, deviant syllable /ga/ presented to the right ear, deviant syllable /pa/ presented to the right ear.

#### 2.11.1 Mood induction

Contrast images at the individual level were computed comparing the three mood induction blocks in each session to the baseline based on the hrf (t-contrast). The three contrast images of the three mood induction conditions were each analyzed with a one-sample t-test at the group level. Only clusters above the cluster-level threshold according to FWE-corrected p<.05 (height threshold T>4.59, extent threshold 50 voxels) were reported.

#### 2.11.2 Deviant events

At the individual level, contrast images were computed according to the factors mood (sad, happy, neutral), presentation side of deviant syllable (left, right) and BOLD response (hrf, temporal derivative), resulting in twelve images per subject. To measure the effects of induced mood and side of deviant on brain activity, a repeated-measures model with condition as the fixed factor and subject as the random factor was applied and the twelve contrast images were implemented as conditions. Inference statistics were based on the effects of interest contrast (F-contrast) at the threshold of FWE-correction (p<.05). Only clusters with a minimal volume of 120 µl (15 voxels) were considered.

For the hypothesis-driven region of interest (ROI) analyses, contrast estimates were extracted from the activation peaks in bilateral auditory cortices. To adjust the hemodynamic response, the generic model function and its derivative were weighted according to the best fit across all deviants. A repeated-measures ANOVA with the factors *mood* (sad, happy, and neutral) and *presentation side* of deviant (left ear, right ear) was conducted. Post-hoc testing disentangled the effects in pair-wise comparisons. To control for sex effects, an ANOVA was computed with the same design but including the intersubject factor gender. Significance level was set at p<.05 for hypothesis testing.

## Results

### 3.1 Behavioral results

#### 3.1.1 Dichotic listening task

All subjects except one showed a higher right-ear than left-ear score, demonstrating an REA (laterality index: mean right-ear decisions minus left-ear decisions 98.9±66.4). Left hemisphere dominance for phonetic processing and language can therefore be assumed in this group.

#### 3.1.2 Mood ratings

**Figure 2:**
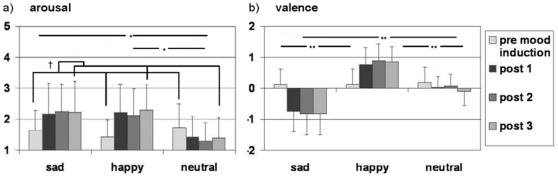
Self-Assessment Manikin (SAM) ratings before and after mood induction (mean±SD). Ratings of a) arousal and b) valence reveal significant effects of mood (*: p<.01; **: p<.001) and time (†: p<.05); pre mood induction: rating prior to first mood induction block, post1/post2/post3: rating after first/second/third mood induction block.

Mood induction was confirmed by significant mood effects on the arousal and valence ratings (Figure 2). The repeated-measures ANOVA for arousal ratings yielded a significant effect of mood (F[2,18] = 6.676, p = .007), time (F[3,17] = 3.763, p = .031), and of the mood x time interaction (F[6,14] = 5.008, p = .006). Pairwise comparisons (mean difference±SE) revealed significantly higher arousal in the sad (0.612±0.169, p = .005) as well as in the happy than the neutral condition (0.556±0.156, p = .006), with no difference between sad and happy. Moreover, the significant time effect emerged between the rating prior to the mood induction and the rating after the third mood induction block (−0.375±0.106, p = .013; Figure 2a).

Valence ratings revealed a significant effect of mood (F[2,18] = 26.794, p<.001) and a significant mood x time interaction (F[6,14] = 8.066, p = .001). Pairwise comparisons (mean difference±SE) showed a significant effect of all pairs of mood conditions: sad and neutral (0.619±0.090, p<.001), happy and neutral (0.613±0.105, p<.001), as well as sad and happy (1.231±0.166, p<.001; Figure 2b).

As concerns the ESR, prior to the first mood induction block, the average rating score was highest for neutral in all three mood conditions, indicating that subjects were predominantly in a neutral mood before each mood induction session (Table 2). After mood induction, the average rating for happiness was highest in the happy condition and highest for neutral in the neutral condition. However, mean scores for sadness and neutrality were the same in the sad condition. Nevertheless, there was a notable increase of the mean score of sadness pre- to post-rating (see Table 2).

**Table 2:** Emotional Self-Rating prior to and after mood induction.

	Sad mood induction		Happy mood induction		Neutral mood induction	
	pre	post	pre	post	pre	post
Fear	1.08±0.18	1.15±0.37	1.13±0.28	1.08±0.18	1.13±0.36	1.08±0.24
Disgust	1.08±0.24	1.20±0.44	1.05±0.22	1.08±0.24	1.10±0.31	1.08±0.24
Happiness	2.50±0.99	1.88±1.06	2.65±1.03	**3.48±1.09**	2.45±0.83	2.18±1.07
Neutrality	**3.43±1.15**	**2.70±1.06**	**3.40±1.30**	2.60±0.95	**3.35±1.36**	**3.98±1.26**
Sadness	1.10±0.26	**2.70±1.30**	1.15±0.33	1.08±0.24	1.25±0.50	1.15±0.33
Anger	1.13±0.43	1.30±0.75	1.15±0.40	1.13±0.32	1.28±0.50	1.18±0.29

The highest rating score for each condition set in bold (mean±SD); pre: prior to the first mood induction block, post: after the final mood induction block.

### 3.2 fMRI results

#### 3.2.1 Mood induction

**Figure 3:**
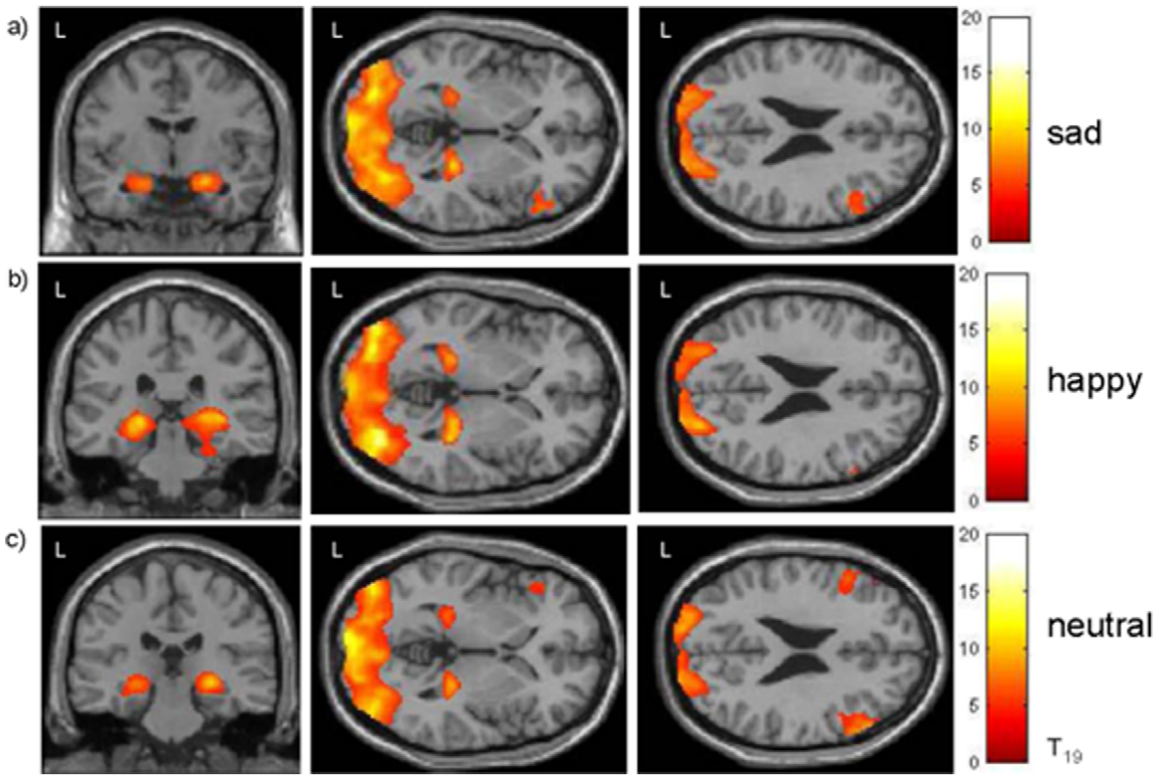
Hemodynamic responses to a) sad, b) happy and c) neutral mood induction. A wide-spread activity in visual areas is due to the procedure using facial presentations. Notable are bilateral amygdala responses and right-lateralized frontal activation during sadness as well as hippocampus responses during happiness, confirming the effectiveness of mood induction independent from the ongoing acoustic stimulation; height threshold T>4.59, extent threshold 50 voxels.

The main effect of all three mood induction conditions achieved by face presentation yielded extended activations in the visual and the limbic system (amygdala and hippocampus; see Table 3 and Figure 3). Additional activation was observed in the prefrontal cortex with a right-lateralized pattern in the sad condition.

**Table 3:** Mood induction networks.

Mood condition	Anatomical region	Hemisphere	BA	MNI coordinates			Peak	Cluster size [voxel]
				X	Y	Z	t-values	
Sad	Middle occipital gyrus	R	19	28	−94	16	16.23	15312
	Thalamus	R		24	−30	−2	10.35	419
	Amygdala	R		24	−6	−18	9.13	450
	Amygdala	L		−24	−2	−22	8.38	981
	Inferior frontal gyrus	R	47	56	30	0	6.43	641
	Superior frontal gyrus	R	9	10	56	44	6.35	76
Happy	Inferior occipital gyrus	R	18	40	−84	−14	17.89	16448
	Parahippocampal gyrus	L	27	−22	−30	−4	10.76	644
	Rectal gyrus		11	0	34	−20	5.28	70
Neutral	Inferior occipital gyrus	L	18	−34	−86	−12	17.58	14229
	Thalamus	R		26	−28	−4	10.90	421
	Middle frontal gyrus	R	46	58	30	22	9.22	819
	Inferior frontal gyrus	L	9	−56	20	28	7.89	638
	Lateral geniculum body	L		−24	−26	−6	7.50	348
	Amygdala	R		18	−6	−20	7.01	158
	Superior frontal gyrus	L	6	−6	34	64	6.45	169
	Superior frontal gyrus	R	9	10	56	44	6.34	304
	Inferior frontal gyrus	R	47	34	24	−22	5.96	96
	Medial frontal gyrus	R	11	2	50	−16	5.95	259

Cluster-level threshold according to FWE-corrected p<.05 (height threshold T>4.59, extent threshold 50 voxels); BA: Brodmann Area, MNI: Montreal Neurological Institute.

#### 3.2.2 Deviant events

**Figure 4:**
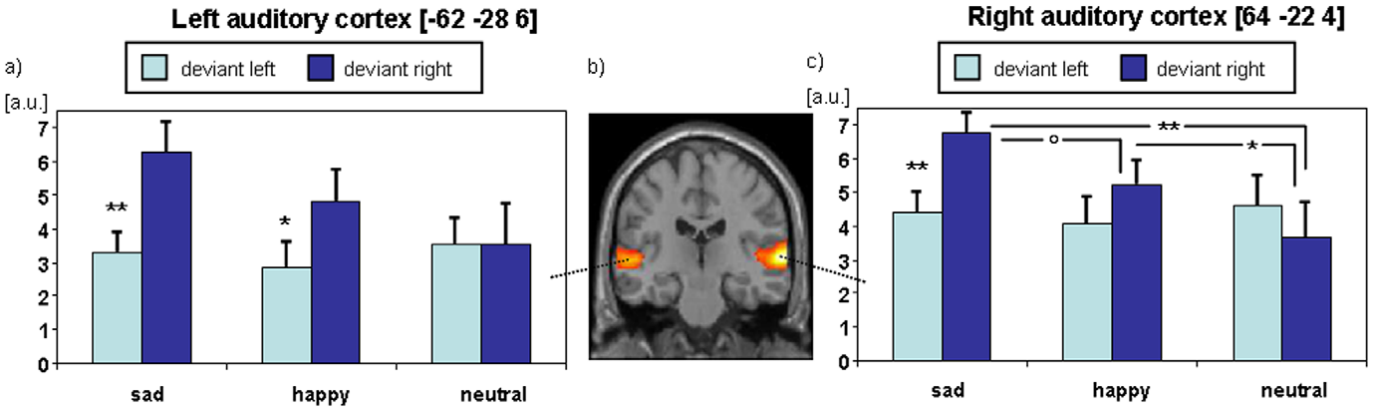
Mapping revealed hemodynamic responses to deviant events at the left and the right auditory cortex (panel b; FWE-corrected p<.05, extent threshold 15 voxels). In the ROI analyses, (a) the responses at the left hemisphere showed a significant effect of presentation side and (c) the right auditory cortex exhibited a significant interaction of mood and presentation side. In particular, right-ear deviants elicited significantly higher activation in the right auditory cortex during sad mood as compared to neutral mood and as compared to left-ear stimuli (**: p<.01; *: p<.05; °: p<.1; mean±SE); a.u.: arbitrary units.

As hypothesized, deviant events yielded a strong hemodynamic response in bilateral superior temporal plane. Bilateral visual cortices were also activated. These bilateral visual clusters survived the conservative FWE-correction, confirming that auditory deviant processing interacted with visual processing (Table 4 and Figure 4).

**Table 4:** Activation clusters to auditory deviants.

Anatomical region	Hemisphere	BA	MNI coordinates			Peak	Cluster size [voxel]
			X	Y	Z	F-values	
Superior temporal gyrus	R	22	64	−22	4	17.99	1427
Superior temporal gyrus	L	22	−62	−28	6	11.54	1057
Lingual gyrus	L	17	−20	−84	−6	7.40	673
Cuneus	R	18	12	−78	12	6.15	113
Lingual gyrus	R	18	24	−80	−6	6.07	104
Middle occipital gyrus	R	18	14	−92	10	5.46	21
Lingual gyrus	L	19	−18	−58	−4	5.42	33

FWE-corrected p<.05, extent threshold 15 voxels; BA: Brodmann Area, MNI: Montreal Neurological Institute.

The main hypothesis stated mood effects on laterality of auditory processing, which were addressed with ROI analyses. A repeated-measures ANOVA with the 3-level factor *mood* (sad, happy, and neutral) and the 2-level factor *presentation side* (left-ear vs. right-ear deviant) assessed the response amplitudes at the activation peaks at the left and right auditory cortices. At the left auditory cortex, a significant effect emerged only for presentation side (F[1,19] = 8.095, p = .010) but not for mood (F[2,18] = 1.335, p = .288). The interaction just failed significance (F[2,18] = 3.257, p = .062). Post-hoc t-tests confirmed larger responses to right-ear deviant syllables in sad and happy mood (mean difference left-right for sad: −2.974±3.768, t[19] = −3.530, p = .002; happy: −1.965±3.435, t[19] = −2.558, p = .019) but not in the neutral condition (0.018±4.579, t[19] = 0.017, p = .986; Figure 4a).

The right auditory cortex responses yielded no significant main effects of mood (F[2,18] = 1.993, p = .165) and presentation side of deviant (F[1,19] = 1.456, p = .242). Importantly, a significant interaction of mood and presentation side emerged (F[2,18] = 4.468, p = .027). As concerns the post-hoc t-tests for presentation side, the same pattern emerged as at the left hemisphere (sad: −2.415±3.287, t[19] = −3.285, p = .004; happy: −1.142±3.696, t[19] = −1.381, p = .183; neutral: 0.920±5.050, t[19] = 0.815, p = .425; Figure 4c), except for neutral mood yielding slightly higher responses to left-ear deviants. The mood-side interaction was characterized by differences in the responses to right-ear deviants; the sad condition yielded higher responses compared to neutral (3.126±4.451, t[19] = 3.141, p = .005) and – on a trend level – to happy (1.568±3.447, t[19] = 2.034, p = .056) as well as happy compared to neutral (1.558±2.769, t[19] = 2.516, p = .021; for left-ear deviants, all p>.2).

The findings were robust against the inclusion of the intersubject factor gender, i.e., the observed effects remained; only the interaction of mood and presentation side at the left auditory cortex barely survived the significance threshold (p = .044, without gender p = .062) reflecting a subtle modulation of variance by the introduced covariate. No significant main effect or interaction with the other predictors emerged (all p>.2, except the interaction of presentation side and gender; left auditory cortex: p = .072, right auditory cortex: p = .055).

## Discussion

The present study examined the influence of induced mood on lateralized processing of acoustic stimuli in the auditory cortex in a group of left-hemisphere dominant healthy volunteers. During task-irrelevant dichotic stimulation, mood induction with emotional facial expressions yielded behavioral effects and activation in brain areas known to be involved in emotion processing, such as amygdala, hippocampus and prefrontal cortex. The phonetic deviants elicited hemodynamic mismatch responses in auditory and visual cortices. A strong modulation of lateralized processing by induced mood was observed. Both emotion conditions (sad and happy mood) yielded a relative preponderance of activation to right-ear deviants in left auditory cortex, whereas in right auditory cortex right-ear deviants elicited higher activation during sad mood, reflecting the interaction of negative emotion processing in the right hemisphere and lateralized auditory processing. Overall, deviant events presented to the right ear elicited strongest activation during sad mood.

### 4.1 Mood induction

Consistent with previous studies, the present results indicate the success of mood induction [24]. Neural activity during mood induction was revealed in visual (occipital pole) and limbic areas (amygdala and hippocampus) in all three conditions (Figure 3). Neural activity in these regions during mood induction has been previously reported [32]–[34]. Bilateral prefrontal cortex activation was found in the neutral mood condition. A right-lateralized pattern of prefrontal activation was found in the sad condition. The laterality of prefrontal cortex activation in sad mood points to the specificity of right-hemispheric processing of negative emotion [11]. Overall, activation patterns show that simultaneously running the auditory oddball paradigm did not inhibit the mood induction effect.

### 4.2 Responses to deviant acoustic stimuli

Deviant events triggered activation in bilateral auditory and visual cortices. Activation of the auditory cortex to non-attended changes in the auditory stream is well established using electroencephalography (EEG: [35]–[37]), magnetoencephalography (MEG: [38]–[40]), intra-cranial recordings [41], and fMRI [42]. Different regional MMN responses have been described – such as in primary auditory cortex, cortical areas in planum temporale and posterior superior temporal gyrus, and ventrolateral prefrontal cortex – and have been associated with different psychophysiological properties [15]. To our knowledge it has not been documented that primary visual cortices are modulated in response to unattended auditory deviants. However, our findings suggest that there is an effect of pre-attentive auditory deviant stimuli on visual cortex activation.

The activated voxels in the visual cortex survived the rather conservative FWE-correction – though exhibiting a smaller effect size than auditory cortex. The mood induction task in the present study has an explicit visual component that requires participants to direct their attention towards the stimuli. Even though more standard MMN paradigms involve reading, watching a movie or even attending to a visual task (e.g. [43]), the present study required complex visual processing and feature extraction for the emotion recognition component. A modulation of attention thus can be expected to alter neural activity in the visual domain. Most significantly, responses in the visual cortex reflect the theorized function of the mismatch response. Näätänen [44] pointed out the putative mechanism of the MMN, theorizing that a mechanism within the early cortical processing helps to involuntarily direct attention to relevant – in this case – changing features of the environment. Such mechanism may work supramodally and result in a higher excitability of the visual and other sensory systems. Considering the ongoing visual stimulation, the observed BOLD response in the visual cortex seems to be a conceivable consequence of an increase in metabolic demand to attention shifts elicited by deviant events. Nevertheless, the latency and duration of these responses cannot be derived from the BOLD signal.

### 4.3 Induced mood modulates responses to deviant acoustic stimuli

The present study investigated how lateralized processing of deviant events is modulated by mood induction. Auditory responses to right-ear deviants were enhanced during sad mood as compared to neutral mood, reflecting mood-dependent modulation of mismatch responses to consonant-vowel syllables. Previous studies investigating the effect of emotional context on the processing of neutral auditory stimuli reported enhanced acoustic novelty processing for negative valence [20]–[23]. In a similar vein, dysphoric persons show impaired attention disengagement from negative stimuli [45], [46]. Thus negative context and content may yield preferential processing of the irrelevant sounds because of higher relevance in danger detection.

The mood induction procedure in the present study yielded similar arousal ratings for happy and sad mood, which were significantly higher than during the neutral condition (Fig. 2) – in contrast to a perception task in which sad faces were rated as low on the arousal dimension (e.g. see [47]). Mismatch responses were significantly stronger during both mood conditions (right ear, right hemisphere; see Fig. 4). Similarly, MMN responses were attenuated in a non-arousing environment because of decreased relevance of potential threats [48]. Moreover, in the present study, auditory activity during the distinct valence conditions differed at a trend level. In a similar vein, Alexandrov and colleagues [20] observed enhanced auditory mismatch responses in negative emotional context, which were not sufficiently explained by arousal as well. In the present study, the enhanced mismatch responses in sad mood may be due to an additive effect of negative valence and increased arousal (see also [48]).

### 4.4 Mood and laterality

An increased REA, which is an explicit measure of laterality, was found in depressive patients [18], [19]. Brain responses were found to support contralateral processing, with a right-ear advantage for language stimuli [49], [50] and frontal involvement in dichotic listening tasks [51]–[53]. In the present study, auditory cortex responses to *task-irrelevant* dichotic stimuli indicated increased processing of right-ear stimuli on the neural level as well. Liotti and Mayberg [4] suggested that processing of negative emotion in depression and induced sad mood interferes with processing of left-lateralized stimuli in the right hemisphere and thus leads to a bias towards stimuli presented on the right. Limbic activation was suggested to suppress inferior parietal and prefrontal cortex activation. The present data show *activation* of prefrontal cortex instead of *deactivation* in the right hemisphere in sad mood as well as increased activation of right auditory cortex to ipsilateral deviants. This right-hemisphere overactivation may serve as a compensatory mechanism to reduce functional impairment of the right hemisphere in depression (for a review, see [54]). In our data, both hemispheres responded stronger to right-ear deviants during induced sadness, but particularly at the right hemisphere these stimuli were processed with increased activity. Therefore, the enhanced excitability of the right auditory cortex to ipsilateral stimuli may reflect a compensatory mechanism in sad mood.

Differences in the procedure may account for some discrepancy to a study that yielded a decreased REA after negative mood induction. Gadea et al. [55] induced negative affect with self-referent statements expressing depressed mood, the Velten Mood Induction Procedure (VMIP; [56]). The authors described that the subjects with an REA in the neutral mood induction showed a reduced REA after induction of negative affect. In the present study, mood induction was conducted with emotional facial expressions and dichotic stimuli were task-irrelevant. Moreover, Gadea and colleagues, pointed out that the induction of negative affect may have enhanced anxiety as well. Indeed, a smaller REA was also observed in depressive patients with comorbid anxiety compared to nonanxious patients [18], [19]. In the present study, right-hemispheric valence and arousal effects seem to foster the processing of right-ear deviant syllables by enhancing auditory cortex excitability to ipsilateral deviant events. Domínguez-Borràs and colleagues [23] suggested an altered excitability in the auditory change detection system in the context of emotional salience.

### 4.5 Limitations and outlook

Subjects were not asked to report strategies for reaching the mood state shown in the emotional facial expressions. Subjects were only instructed to try to feel the mood seen on the pictures. One might argue that watching facial expressions of emotions only elicits perceptual processing of emotion rather than feeling the emotion. Mood ratings, however, indicated that arousal and valence changed according to the emotion presented and thus successful mood induction can be assumed. Ideally, an independent measure of mood would have been optimal to rule out any social desirability effect [24].

Whereas functional magnetic resonance imaging provides high spatial resolution, the temporal resolution is very poor compared to electrophysiological measures. As such, we cannot draw conclusions about latency and duration effects of the auditory cortex responses. On the other hand, without the higher spatial resolution in fMRI, we would not have been able to reveal the visual cortex activation to deviant events. Given that the laterality of mismatch responses to auditory stimuli follows a time course [57], [58], we cannot rule out that the right-lateralized pattern in the present study is due to a latency effect. Indeed, for MEG recordings during pitch identification, a faster right-hemispheric response has been found according to the left-ear advantage for pitch processing, but the categorization task was left-hemispheric [43]. In the present study, the larger right-hemispheric responses may be due to the emotional task and be only apparent at higher latency. These latency effects cannot be disentangled by means of fMRI.

Gender effects in emotion processing (see for example [59]) may be of particular interest in the modulation of mismatch responses. In the present study, the introduction of gender as an intersubject variable did not yield any significant main effect or interaction effect but may have reflected a subtle influence on laterality. However, our sample was not balanced and we did not control for menstrual cycle to directly address this question. In general, sex effects on mismatch negativity are still under debate [60], [61].

### 4.6 Conclusion

The present study demonstrated an influence of induced mood states on auditory cortex activation to dichotically presented deviant syllables. Prefrontal top-down influences on auditory processing may be the underlying mechanism leading to enhanced processing of right-ear deviants. Moreover, compensatory resource allocation to the right hemisphere in the context of negative valence was reflected in higher excitability of the right auditory cortex to ipsilateral deviants. Present findings emphasize the role of cognitive and emotion lateralization by indicating a strong mood-dependent modulation of lateralized auditory processing, even in the absence of voluntarily directed attention to spatially presented stimuli. Ipsilateral processing may account for the enhanced right-hemispheric response to right-ear deviants during mood changes.
